# N6-methyladenosine Modification of Noncoding RNAs: Mechanisms and Clinical Applications in Cancer

**DOI:** 10.3390/diagnostics12122996

**Published:** 2022-11-30

**Authors:** Mingyang Ma, Tong Ye, Jiewei Wang, Haiying Zhao, Shutian Zhang, Peng Li, Guiping Zhao

**Affiliations:** Department of Gastroenterology, Beijing Friendship Hospital, Capital Medical University, Beijing 100050, China

**Keywords:** N6-methyladenosine, noncoding RNA, cancer

## Abstract

N6-methyladenosine (m6A) modification remains the most pivotal epigenetic modification on RNA. As we know, m6A not only affects physiological processes but is also involved in carcinoma. Noncoding RNAs play an indispensable role in the occurrence and development of carcinoma. However, a large amount of research is focused on mRNA currently. Insufficient research has been done on the relationship between noncoding RNA (ncRNA) methylation and cancer. Therefore, this review aims to introduce the theoretical knowledge of m6A modification in noncoding RNA, discuss its function in tumorigenesis and progression, and ultimately summarize its potential clinical applications.

## 1. Introduction

The central dogma declares that DNAs are transcribed into RNAs which are translated into proteins thereafter [1]. However, with the advent of the human gene program in June 2000, we realized that only a few genomes of humans encode for proteins via the transcription of mRNA. Scientists started to dig into the function and mechanism of noncoding RNAs (ncRNAs), including micro RNAs (miRNA), small nucleolar RNAs (snoRNAs), long noncoding RNAs (lncRNA), small nuclear RNAs (snRNAs), and circular RNAs (circRNA) [2,3,4]. In contrast to mRNA, ncRNAs cannot encode proteins, although they are involved in epigenetic modifications and gene-transcription regulation at the RNA level [3].

The concept of N6-methyladenosine (m6A) was first reported by several revolutionary studies in the 1970s [5,6]. The process of m6A methylates adenosine at the N6 position, which is common in both coding and noncoding RNAs, acting as the most common process of methylation of RNAs until now [7]. In 1997, scientists successfully cloned the first m6A writer, methyltransferase-like protein 3 (METTL3), which methylates nearly all m6A in mRNA [8]. In 2011, the fat mass and obesity-related protein (FTO) was identified as the first m6A demethylase, suggesting that m6A modification is reversible and dynamic, which injected new vitality into epigenetics [9].

Though discovered in the 1970s, the explicit mechanism of how m6A affects gene transcription and translation was vague until the development of m6A-mapping methods such as m6A individual-nucleotide-resolution crosslinking and immunoprecipitation (miCLIP), methylated RNA immunoprecipitation sequencing (Me-RIP seq), and DNA-ligation detection [10,11,12]. Due to these techniques, m6A modification on ncRNAs was proven to engage in the etiopathology of many diseases, including cancer.

## 2. Regulators of m6A

### 2.1. m6A Writers

There are several methyltransferases related to m6A modification in both coding and noncoding RNA. MTC, m6A methyltransferase complex, consists of methyltransferase-like protein 3/methyltransferase-like protein 14 heterodimer (METTL3/METTL14), Wilms tumor 1 associated protein (WTAP), zinc finger CCCH-type containing 13 (ZC3H13), vir-like m6A methyltransferase-associated (VIRMA), Cbl proto-oncogene like 1 (CBLL1, or Hakai), and RNA-binding motif 15 or its homologous RBM15B (RBM15/15B) [13,14]. METTL3/METTL14 installs methyl group on adenosines in a designated sequence RRACH (R = A or G; H = A, C, or U). METTL3 is the catalytic part of METTL3/METTL14 [15,16]. By contrast, METTL14 has no methyltransferase activity and plays the role of an allosteric activator [15,17]. However, once combined with each other, METTL3 and METTL14 have an increase in the efficiency of transferring a methyl group from the donor substrate S-adenosyl methionine (SAM) to RNAs [17]. The rest of the MTC subunits perform auxiliary functions. For instance, WTAP assists MTC in anchoring to chromatin. VIRMA mediates m6A methylation in the 3′UTR by recruiting polyadenylation specificity factors and is associated with selective polyadenylation. ZC3H13 bridges between RBM15/15B and WTAP and executes its duty to help MTC locate in the nucleus [18,19,20].

METTL16 is another writer found in both nuclear and cytoplasm which has been demonstrated to methylate U6 snRNA [21,22]. Notably, it is reported that METTL16 could methylate the long noncoding RNAs (lncRNA) MALAT1 and XIST [23]. In addition to the writers mentioned above, proteins like METTL5, TRMT112, and ZCCHC4 also play important roles in the process of N6-methylation.

### 2.2. m6A Erasers

The course of m6A modification is reversible because of two demethylases, fat mass and obesity-associated protein (FTO) and AlkB homolog 5 (ALKBH5). Identified as the first RNA demethylase, FTO is a member of the FeII/α-KG-dependent dioxygenase AlkB family and removes m6A and N6,2-O-dimethyladenosine (m6Am) in vitro and in vivo [9,24]. It came as a milestone in the domain of epigenetics and strengthened scientists’ confidence that m6A modification is dynamic. Consequently, ALKBH5, another eraser demethylating m6A exclusively, was identified two years later [25]. The nucleotide recognition lid (NRL) structure in ALKBH5 is responsible for m6A recognition and methylation catalysis [26]. Moreover, AlkB homolog 3 (ALKBH3), another member of AlkB family, is shown to increase translation productivity via catalyzing m6A demethylation on tRNA [27]. However, albeit the contribution to the reversible process of m6A removal, m6A erasers only exist in some definitive tissues and certain circumstances related to emergencies or stress [28].

### 2.3. m6A Readers

m6A readers, groups of m6A-binding proteins which include the YT521-B homology (YTH) domain family, heterogeneous nuclear ribonucleoproteins (HNRNPs), and insulin-like growth factor 2 mRNA-binding proteins (IGF2BPs), conduct the function of m6A in post-transcriptional gene modification by recognizing and combining m6A on RNAs or changing the structure of RNAs [29,30,31,32,33,34,35].

#### 2.3.1. YT521-B Homology (YTH) Domain Family

The YT521-B homology (YTH) domain family, which contains YTHDC1, YTHDC2, YTHDF1, YTHDF2 and YTHDF3, binds m6A with the YTH domain directly [29]. YTHDC1 preferentially identifies m6A on the X-inactive specific transcript (XIST) of long noncoding RNA (lncRNA) to engage in the transcriptional silencing of the X chromosome [36]. In mRNA, YTHDC1 facilitates exon inclusion by recruiting pre-mRNA splicing factor SRSF3 and disturbing the combination between SRSF10 and mRNA [37]. YTHDC2 is abundant in testicular cells. Although it has been studied for many years, the full mechanism of YTHDC2 is still unclear. Different from YTHDC1, YTHDC2 binds weakly to m6A on RNAs [38]. YTHDF2 was the first confirmed reader. The N-terminal region of YTHDF2 interplays with the SH domain of CNOT1, a subunit of CCR4-NOT, inducing the degradation of m6A-modified RNA by recruiting CCR4-NOT deadenylase complex [30]. YTHDF3 promotes protein translation together with YTHDF1 and affects methylated mRNA degradation via YTHDF2 [31]. YTHDF1, YTHDF2, and YTHDF3 go through liquid-liquid phase separation in vivo and in vitro. Polymethylated mRNA combines with YTHDF proteins and functions as a carrier. Then mRNA-protein complex is segmented into various compartments, such as P-bodies and stress granules. Notably, a study found the number and location of m6A sites on mRNA affect the composition of the complex and liquid-liquid phase [39].

#### 2.3.2. Heterogeneous Nuclear Ribonucleoproteins

Heterogeneous nuclear ribonucleoproteins (HNRNPs) include HNRNPC, HNRNPA2B1, and HNRNPG. HNRNPC is an RNA-binding protein enriched in pre-mRNA processing. The M6A-U base pair is unstable because the binding of an m6A-U base pair is weaker than that of an A-U pair. m6A contributes to the unfolding form of lncRNA and mRNA, therefore exposing buried binding points and increasing the accessibility of readers to m6A-RNA. This mechanism is called “the m6A-switch” [40]. As a result, HNRNPC can be easily bound to the RNA sites and performs its duties [34]. The m6A-switch mechanism also facilitates the binding of the other two HNRNPs with RNA: HNRNPA2B1 and HNRNPG. The former is an RNA-binding protein that promotes primary miRNA processing [35] and the latter is another HNRNP that regulates the expression and the splicing process of target mRNAs [41].

In addition to the readers mentioned above, several RNA binding proteins can also mediate gene expressions, such as insulin-like growth factor 2 mRNA-binding proteins 1/2/3 (IGF2BP1/2/3) and Fragile X mental retardation RNA binding protein (FMRP). The mechanism of how readers choose the binding sites on RNA is still not clear by now. Here are three hypotheses: First, readers may choose a unique site on RNA by interplaying with other readers to identify the intrinsic characters of RNA [42]. Secondly, the enrichment of m6A regions denotes more opportunities for readers to encounter them. In addition, the distinct array of RNA sequences induces the binding of readers with certain RNA transcripts. For example, FMR1 is a typical sequence-array-based reader which could regulate RNA translation [43]. Finally, reader proteins may gather in specific cellular components in a heterologous way. For instance, stress granules (SGs) are RNP granules synthesized once a cellular stress condition occurs. Being a part of the SG proteome, YTHDF1-3 forms clusters in SG core clusters [44]. The function of regulators is summarized in Table 1.

## 3. m6A-Modification and ncRNA

### 3.1. m6A-Modification in miRNA

miRNAs are short ncRNAs with an average length of 22 nt. A mature miRNA derives from a primary miRNA (pri-miRNA) which is cleaved by RNA Polymerase II (Pol II) from DNA. Then, the pri-miRNA is processed into a precursor miRNA (pre-miRNA) with the assistance of a microprocessor complex, consisting of Drosha, a ribonuclease III enzyme, and DiGeorge Syndrome Critical Region 8 (DGCR8) [62,63]. The lack of METTL-3 is proven to affect miRNA biogenesis by reducing the binding between DGCR8 and pri-miRNA [64]. As a result, the number of mature miRNAs decreases, and pri-miRNAs without cleavage accumulate.

In further studies, METTL-3 was found to accelerate the maturation of pri-miR221/222 via interplaying with DGCR8, leading to the oncogenesis of bladder cancer [65]. In addition, METTL-3 was revealed to promote the modification of miR-181d-5p in some patients with resistance to 5-Fluorouracil (5-FU), which was attributed to the interaction between METTL-3 and DGCR8 [66]. Besides DGCR8, METTL-3 was reported to engage in the axis of METTL3-miR-25-3p-PHLPP2-AKT as an outcome of smoking. Cigarette smoke condensate (CSC) triggers the overexpression of METTL-3, which catalyzes the methylation thereafter being decoded by an m6A reader, NF-κB associated protein (NKAP). Methylation could affect the maturity of miR-25-3p, then prohibit PH domain leucine-rich repeat protein phosphatase 2 (PHLPP2), which provokes the oncogenic signaling of AKT-p70S6K and finally causes pancreatic cancer [67]. In hepatocellular carcinoma (HCC), METTL-14 can also interact with DGCR8. Downregulation of METTL-14 inhibits the expression of miRNA 126 (a cancer suppressor) and gives rise to the proliferation of HCC [68].

Recently, FTO was discovered to increase the expression of ADP ribosylation factors like GTPase 5B (ARL5B) in breast cancer cells by inhibiting miR-181b-3p. MiR-181b-3p is an miRNA that takes part in the FTO/miR-181b-3p/ARL5B axis [69]. Yes-associated protein (YAP) is an important transcriptional regulator controlled by m6A modification on miRNA in cancer progression. Overexpression of ALKBH5 reduces the expression of miR-107. However, silencing depends on Human antigen R (HuR), which can reverse this process and decrease YAP activity by intervening in the axis of miR-107/LATS2 depending on HuR, which indicates ALKBH5 is a suppressing factor in the oncogenesis of nonsmall-cell lung cancer (NSCLC) [70,71].

RALY (HNRNPCL2) is newly found as an RNA-binding protein associated with the aggressiveness of colorectal cancer (CRC). As a part of the Drosha complex, RALY processes the maturation of a group of miRNAs, such as miR-483, miR-676, and miR-877. Consequently, these miRNAs downregulate relative genes in the mitochondria and contribute to the growth of CRC cells [72]. HNRNPA2B1 interacts with DGCR8 to promote the generation of pre-miRNA [33]. In tamoxifen-resistant breast cancer cells, HNRNPA2B1 was reported to be upregulated, which led to the hypothesis that HNRNPA2B1 facilitates endocrine resistance by affecting the expression of miRNA. A follow-up study revealed that the overexpression of HNRNPA2/B1 reduced miR-29a-3p, miR-29b-3p, and miR-222. On the contrary, miR-1266-5p, miR-1268a, and miR-671-3p were upregulated in MCF-7 cells [73]. The result manifests the overexpressed HNRNPA2B1 and contributes to the reduction of certain miRNA, which leads to tamoxifen resistance in breast cancer cells.

The evidence above confirms that m6A-modification plays an important part in miRNA in the occurrence and development of cancer. However, further studies are still needed.

### 3.2. m6A-Modification in lncRNA

lncRNAs are ncRNA transcripts with at least 200 nucleotides that interact with RNA and proteins to regulate transcription and epigenetic modifications in carcinogenesis [74].

X inactive-specific transcript (Xist) is a regulatory site located on the X chromosome which can produce a lncRNA 15 to 17 kb in length [75]. Knockdown of METTL14 reduces m6A modification on Xist and enhances the expression of Xist which consequently promotes the metastasis and aggressiveness of CRC. Intriguingly, Xist methylated with m6A can be recognized by YTHDF2, resulting in the degradation of Xist [76]. The evidence further confirms the function of Xist is negatively correlated to METTL14 and YTHDF2. In gastric cancer (GC) cells, THAP7-AS1 is a lncRNA which obviously upregulated compared with normal gastric cells. METTL3-mediated m6A modifies the activation of THAP7-AS1 by SP1. SP1 is a transcription factor that carries out the function of oncogenesis and supports the characteristic of THAP7-AS1 in facilitating lymphatic metastasis. Notably, this process is not only modulated by METTL3 but also with the engagement of IGF2BP1 to decipher the signal of m6A methylation [77]. In NSCLC, ABHD11-AS1, a lncRNA, is upregulated. MeRIP-Seq proved ABHD11-AS1 had specific methylation sites which were prone to be installed with m6A by METTL3. As a result, METTL3 induces the upexpression of ABHD11-AS1, thus promoting the proliferation of NSCLC tissue [78]. Another study analyzed the interaction between LNCAROD, an oncogenic lncRNA, and METTL3/METTL14. METTL3/METTL14-induced m6A methylation stabilized LNCAROD in head and neck squamous cell carcinoma (HNSCC) tissue, which augmented malignant cell multiplication and invasion in vivo and in vitro [79]. In nasopharyngeal carcinoma (NPC), WTAP fine-tuned by KAT3A-mediated H3K27 acetylation is required to stabilize the m6A methylation of DIAPH1-AS1, a lncRNA, and plays an essential role in the growth and metastasis of NPC [80].

As for m6A erasers, FTO is reported to demethylate m6A on LINC00022, a lipogenesis-related lncRNA, leading to the upregulation of LINC00022 in esophageal squamous cell carcinoma (ESCC) cells [81,82]. Though progress has been made in discovering the regulation of ESCC, the mechanism of m6A modification in lncRNA cancer susceptibility candidate 15 (CASC15) is poorly identified. A study revealed FTO mediated the demethylation process on CASC15. The absence of FTO impaired neoplastic proliferation and prompted apoptosis of ESCC cells conducted by CASC15 [83], which demonstrated the interaction between erasers and lncRNA in oncogenesis.

Additionally, in pancreatic cancer, IGF2BP2 was reported to combine with LncRNA-PACERR. LncRNA-PACERR is a stimulator of protumor macrophages to increase the stability of KLF12 and c-myc [84] which denotes the critical role of m6A regulators in the polarization of tissue-associated macrophages (TAMs). Of note, besides direct interplay with m6A regulators, lncRNA can encode a peptide with 71 amino acids called RNA-binding regulatory peptide (RBRP) to indirectly bind to IGF2BP1. Therefore, IGF2BP1 recognizes m6A on RNAs and causes tumors [85].

### 3.3. m6A-Modification in circRNA

circRNAs are covalently closed RNAs produced through the back-splicing of exons in eukaryotes. Unlike linear RNAs, circRNAs are unique both in structure and biomedical functions, such as transcription, splicing, and translation [86]. Currently, studies have proved that m6A modification can regulate circRNAs via m6A writers, erasers, and readers and vice versa [87,88,89]. There are four main aspects that m6A interacts with circRNAs: (1) m6A regulates the biogenesis of circRNAs. In NSCLC, circIGF2BP3 is methylated with m6A by METTL3 and circulated in a YTHDC1-dependent manner to impair the immune response of cancer [90]. (2) m6A prompts the nuclear exportation of circRNAs. circNSUN2, a circRNA upregulated in liver-metastatic colon cancer, can be exported from the nucleus to cytosol with the presence of YTHDC1. Additionally, METTL3 is found to facilitate the transfer of circNSUN2 [91]. (3) m6A modulates the translation of circRNAs. Different from other ncRNAs, circRNAs are capable to encode proteins driven by m6A modification. The process is mediated by YTHDF3 and enhanced by METTL3/14. M6A-driven circRNA translation was widespread [92]. (4) m6A is associated with the degradation of circRNA. In gefitinib-resistant cells, m6A-modified circASK1 is downregulated due to the increase of endoribonucleolytic cleavage induced by YTHDF2 [93].

**Figure 1:**
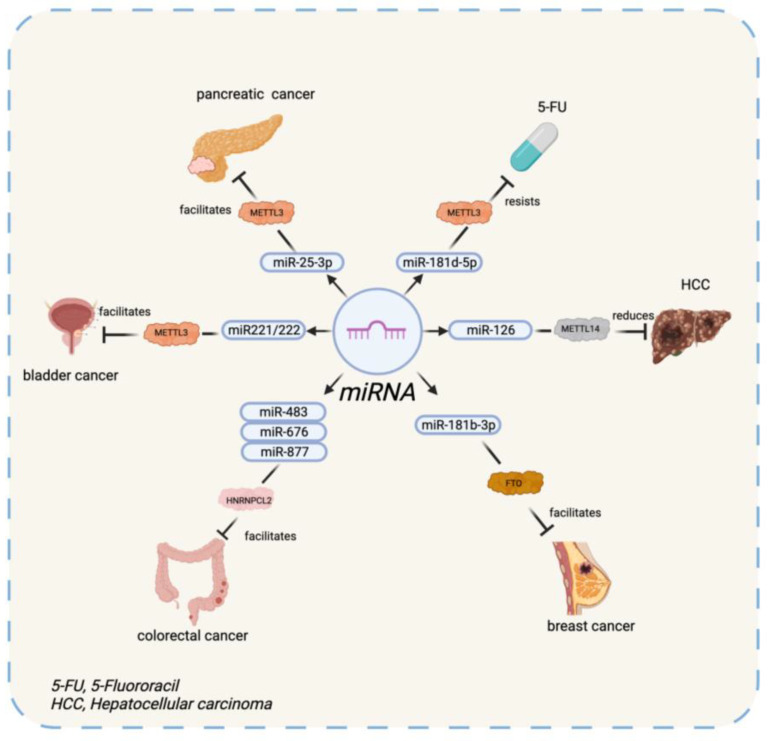
The interaction between miRNA and its m6A regulators.

**Figure 2:**
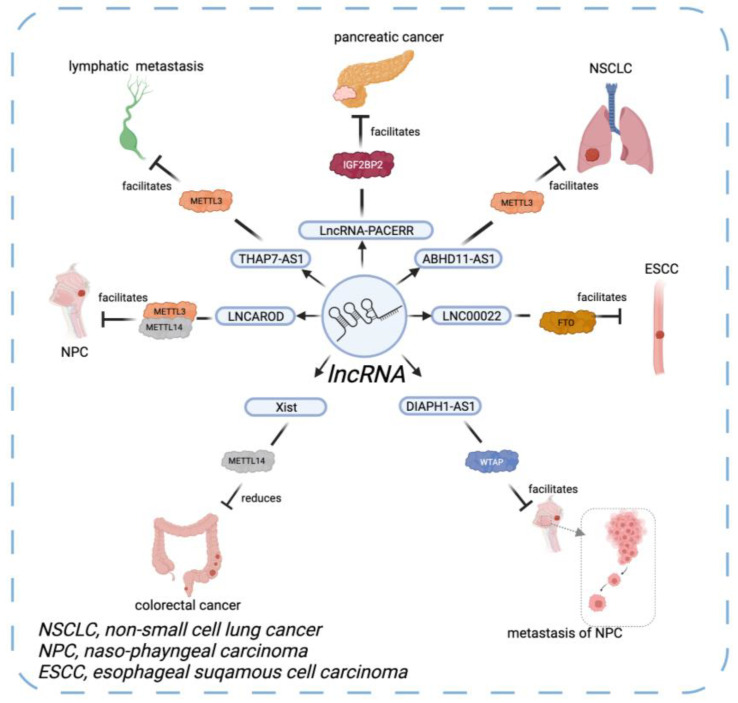
The interaction between lncRNA and its m6A regulators.

**Figure 3:**
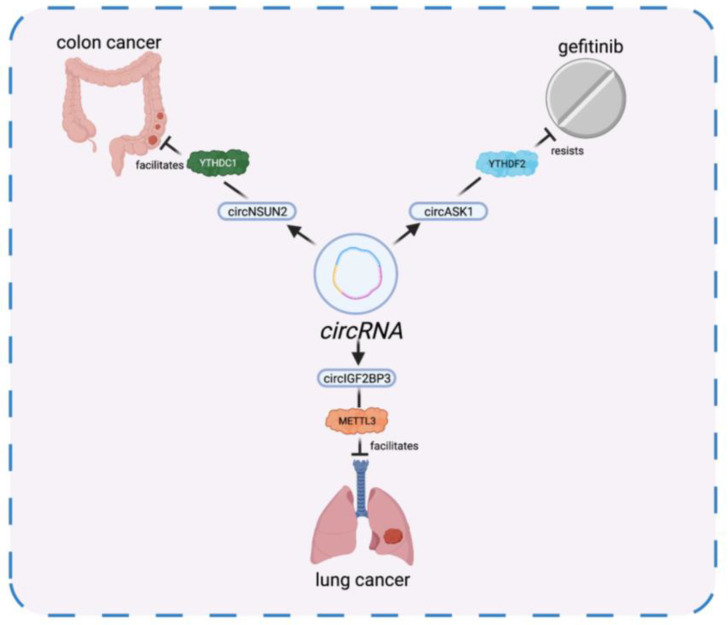
The interaction between circRNA and its m6A regulators.

Notably, the expression and functions of m6A writers, erasers, and readers can be influenced by circRNAs. For example, circRNA can play the role of a miRNA sponge to regulate the expression of YTHDF1 [88]. In addition, ircZbtb20, another circRNA, recruits ALKBH5 to demethylize the m6A on mRNA [94]. To summarize, circRNAs widely interact with m6A in oncogenesis throughout its lifespan. The interaction between ncRNA and their m6A regulators in oncogenesis is summarized in Table 2 and Figure 1, Figure 2 and Figure 3.

## 4. Clinical Applications of m6A for ncRNA in Cancer

Noncoding RNA methylation is closely associated with the occurrence and progression of cancer. However, most of the research still stays in the laboratory stage, and clinical applications were insufficient. This part summarizes the relevant literature regarding its clinical application, hoping to provide new ideas for the diagnosis and treatment of cancer.

### 4.1. Role of ncRNA Methylation in Predicting Prognosis of Cancer

Noncoding RNA methylation can be used as a marker to predict prognosis. For example, overexpression of miR-25-3p caused by METTL3 indicates a poor survival of pancreatic cancer [67]. METTL3 increases the biogenesis of miR-143-3p, which was negatively correlated with the overall survival (OS) rate of lung cancer [95]. M6A may upregulate the expression of LNCAROD by enhancing its stability. LNCAROD increases the expression of pyruvate kinase isoform PKM2 to activate glycolysis in hepatocellular carcinoma cells, eventually elevating the malignancy of HCC. Thus, LNCAROD indicates a poor prognosis for HCC patients [96]. The m6A level of the long noncoding RNA NEAT1 was a powerful predictor of eventual death [97]. Furthermore, METTL3 increases the stability of Lung Cancer Associated Transcript 3 (LCAT3). LCAT3 is known as a long noncoding RNA. Upregulation of LCAT3 represents the poor prognosis of lung adenocarcinoma (LUAD) patients [98]. In addition, Circ3823 facilitates metastasis and angiogenesis of CRC via the circ3823/miR-30c-5p/TCF7 axis. In CRC patients, Circ3823 indicated a worse prognosis [99]. METTL3 promotes the maturation of pri-miR221/ 222 and was associated with poor prognosis in bladder cancer patients [65].

METTL-14 inhibits the expression of miRNA 126, and downregulation of METTL-14 is an adverse prognosis factor in hepatocellular carcinoma [68]. METTL14 downregulates the expression of long noncoding RNA XIST depending on YTHDF2. Decreased expression of METTL14 was associated with an unfavorable prognosis for CRC patients [76]. In gastric cancer, lower expression of METTL14 is associated with poor prognosis. Mechanically, reduced expression of METTL14 upregulated the expression of circORC5. circORC5 could sponge miR-30c-2-3p and downregulate AKT1S1 and EIF4B. Meanwhile, upregulation of circORC5 represents poor prognosis too [100].

The FTO/miR-181b-3p/ARL5B axis modulates the migration and invasion of breast cancer cells. Overexpression of FTO predicts poor prognosis and advanced TNM stage [P = 0.001] [69].

Higher expression of LINC00460 represents poor disease-free and five-year overall survival. LINC00460 interacts with IGF2BP2 and DHX9 to enhance the stability of high-mobility group AT-hook 1 (HMGA1) mRNA [101]. LINC00266-1 encodes RBRP which could interact with IGF2BP1. A high level of RBRP was correlated with poor prognosis [85]. RAL Y is known as a novel m6A reader. Overexpression of RAL Y is associated with a poor prognosis of colorectal cancer [72].

With the advancement of detection technologies, noncoding RNA can be utilized as a reliable indicator to evaluate the prognosis of various cancer.

### 4.2. m6A Inhibitors as Potential Treatment and Diagnostic Target for Cancer

The complex interplay between lncRNA and cancer provides a promising idea for a therapeutic invention for cancer. Moreover, ncRNA may become novel noninvasive diagnosis biomarkers for early detection.

Rhein is known as the first cell-active FTO inhibitor [102] and was demonstrated to inhibit the occurrence of breast cancer [103]. However, the selectivity of Rhein was not optimistic [104]. Meclofenamic acid (MA) is a more specific FTO inhibitor than ALKBH5 [105,106]. MA is able to restrict the growth and self-renewal of glioblastoma stem cells [107]. Furthermore, MO-I-500 was found to be a selective FTO inhibitor, which restrains the survival and colony formation of breast cancer cells [108]. R-2-hydroxyglutarate (R-2HG) is a metabolic product of mutant IDH1/2 which exert its function by inhibiting the m6A demethylase activity of FTO to suppress the progression of leukemic cells [109]. Moreover, FB23-2 is also an FTO inhibitor that significantly restrains the progression of AML cells [110].

In addition to FTO inhibitors, plenty of ALKBH5 inhibitors have been discovered. MV1035 is one of the inhibitors of ALKBH5 which suppress the migration and aggressiveness of glioblastoma [111]. As an inhibitor for METTL3, STM2457 is effective in the inhibition of AML [112,113,114].

Overall, m6A inhibitors offer a new direction for the treatment and diagnosis of different cancers. However, the clinical application of these inhibitors is still insufficient. The efficacy along with the adverse effect of these inhibitors needs further validation.

## 5. Conclusions

In summary, noncoding RNA methylation plays an important role in tumorigenesis and progression. ncRNA can be used as a target for tumor diagnosis and treatment. Based on the current progression and dilemma, we speculate that future research directions could be focused on the following points: Firstly, there is an urgent need to explore novel m6A regulators in-depth, especially whether there is a regulator dedicated to ncRNA. Secondly, further investigation is needed regarding whether there is a link between ncRNA and mRNA methylation. Further research is needed to depict the complex network constituted by both of them in the process of carcinoma. Finally, the clinical application of ncRNA methylation in tumors is inadequate. ncRNA with clinically value still need to be probed. The safety and efficacy of m6A inhibitors still needs to be evaluated in the future.

## Figures and Tables

**Table 1:** A summary of writers, erasers, and readers in RNA m6A-modification.

	Enzyme	Location	Function	Ref.
Writers	METTL3	nucleus and cytoplasm	a catalytic subunit of METTL3/METTL14 dipolymer	[28,45]
	METTL14	nucleus	a platform for METTL3 in the process of RNA recognition and catalyzation	[13,45]
	METTL16	nucleus and cytoplasm	A catalytic subunit methylating U6 snRNA, MALAT1, and XIST	[21,46]
	ZC3H13	nucleus	bridges between RBM15/15B and WTAP and promotes the localization of MTC in the nucleus	[20]
	VIRMA	nucleus	recruits METTL3/METTL14/WTAP to catalyze selective methylation on specific region of RNA	[18]
	WTAP	nuclear speckle	mediates the localization of METTL3 and METTL14 into nuclear speckles	[47]
	HAKAI	-	stabilizes the methyltransferase complex	[48]
	RBM15/15B	-	mediates m6A formation in XIST	[36]
	METTL5	nucleolus and synapse	an enzyme mediating 18S rRNA m6A modification	[49,50]
	TRMT112	nucleus	a methyltransferase activator attaching to METTL5 to mediate m6A modification	[51,52,53]
	ZCCHC4	nucleolus	an enzyme mediating 28S rRNA m6A modification	[54,55]
Erasers	FTO	nucleus and cytoplasm	demethylates m6A unspecifically	[24,28]
	ALKBH5	nucleus	demethylates m6A via oxidation	[24,28]
	ALKBH3	nucleus and cytoplasm	catalyzes m6A demethylation on tRNA	[27]
Reader	YTHDF1	cytoplasm	YTHDF1, YTHDF2, and YTHDF3 act together to induce the degradation of mRNA	[56]
	YTHDF2	cytoplasm		[56]
	YTHDF3	cytoplasm		[56]
	YTHDC1	nucleus	binds to noncoding RNAs like XIST to repress transcription	[57,58]
	YTHDC2	nucleus and cytoplasm	promotes RNA translation; predominately mediates the degradation of mRNA	[38,59,60]
	HNRNPA2B1	nucleus	promotes primary miRNA processing	[33,35]
	HNRNPG	nucleus	regulates the expression and the splicing process of objective mRNAs	[41]
	HNRNPC	nucleus	binds to flanking sequence of RNA to engage in precursor mRNAs (pre-mRNAs) splicing	[34]
	IGF2BP1/2/3	nucleus and cytoplasm	promotes the stability of mRNA under both normal and stress conditions	[42]
	FMRP	nucleus and cytoplasm	stabilizes mRNA via m6A-modification	[61]

**Table 2:** The relationship between ncRNA and m6A regulators in oncogenesis.

Related ncRNA	Regulator Name	Function	Mechanism	Ref.
miRNA	METTL3	1. promotes oncogenesis	① causes bladder cancer by accelerating maturation of pri-miR221/222 via interplaying with DGCR8	[65]
			② causes pancreatic cancer by affecting maturation of miR-25-3p and subsequently prohibits PH domain leucine-rich repeat protein phosphatase 2 (PHLPP2) which provokes AKT-p70S6K	[67]
		2. facilitates resistance to 5-Fluorouracil (5-FU)	interacts with DGCR8 to modify miR-181d-5p	[66]
	METTL14	suppresses oncogenesis	reduces hepatocelluar carcinoma by prohibiting the expression of miRNA 126	[68]
	FTO	promotes the expression of GTPase 5B (ARL5B) in breast cancer cells	Inhibits miR-181b-3p in the FTO/miR-181b-3p/ARL5B axis	[69]
	ALKBH5	suppresses oncogenesis	Reduces the expression of miR-107 in the oncogenesis of nonsmall-cell lung cancer (NSCLC)	[70,71]
	RALY(HNRNPCL2)	promotes oncogenesis	causes colorectal cancer (CRC) by processing maturation of miR-483, miR-676, and miR-877	[72]
	HNRNPA2B1	facilitates resistance to tamoxifen in breast cancer	reduces the expression of miRNA	[33]
lncRNA	METTL3	promotes oncogenesis	① promotes lymphatic metastasis by activating THAP7-AS1	[77]
			② causes NSCLC by promoting the expression of ABHD11-AS1	[78]
	METTL14	suppresses oncogenesis	reduces CRC by facilitating m6A modification on Xist	[76]
	METTL3/METTL14	promotes oncogenesis	causes nasopharyngeal carcinoma (NPC) by stabilizing LNCAROD	[79]
	WTAP	promotes oncogenesis	promotes growth and metastasis of NPC by stabilizing m6A methylation of DIAPH1-AS1	[80]
	FTO	promotes oncogenesis	causes the upregulation of LINC00022 in esophageal squamous cell carcinoma (ESCC) cells	[81,82,83]
	IGF2BP1	promotes oncogenesis	indirectly causes tumor by recognizing m6A on mRNA with the assistance of lncRNA-decoded protein	[85]
	IGF2BP2	promotes oncogenesis	causes pancreatic cancer by combing with LncRNA-PACERR	[84]
circRNA	METTL3	promotes oncogenesis	causes NSCLC by impairing immune response of cancer via methylating circIGF2BP3	[90]
	YTHDC1	promotes oncogenesis	causes the transfer of circNSUN2 from nucleus to cytoplasm in liver-metastatic colon cancer	[91]
	YTHDF2	facilitates resistance to gefitinib	Induces endoribonucleolytic cleavage to downregulate m6A-modified circASK1	[93]

## Data Availability

Not applicable.
